# Automated seeding of specialised wiki knowledgebases with BioKb

**DOI:** 10.1186/1471-2105-10-291

**Published:** 2009-09-16

**Authors:** Jonathan R Manning, Ann Hedley, John J Mullins, Donald R Dunbar

**Affiliations:** 1 Centres for Cardiovascular Science and Inflammation Research, Queen's Medical Research Institute, University of Edinburgh, EH16 4TJ, UK

## Abstract

**Background:**

Wiki technology has become a ubiquitous mechanism for dissemination of information, and places strong emphasis on collaboration. We aimed to leverage wiki technology to allow small groups of researchers to collaborate around a specific domain, for example a biological pathway. Automatically gathered seed data could be modified by the group and enriched with domain specific information.

**Results:**

We describe a software system, BioKb, implemented as a plugin for the TWiki engine, and designed to facilitate construction of a field-specific wiki containing collaborative and automatically generated content. Features of this system include: query of publicly available resources such as KEGG, iHOP and MeSH, to generate 'seed' content for topics; simple definition of structure for topics of different types via an administration page; and interactive incorporation of relevant PubMed references. An exemplar is shown for the use of this system, in the creation of the RAASWiki knowledgebase on the renin-angiotensin-aldosterone system (RAAS). RAASWiki has been seeded with data by use of BioKb, and will be the subject of ongoing development into an extensive knowledgebase on the RAAS.

**Conclusion:**

The BioKb system is available from http://www.bioinf.mvm.ed.ac.uk/twiki/bin/view/TWiki/BioKbPlugin as a plugin for the TWiki engine.

## Background

The storage, collation, and presentation of biological knowledge is a ubiquitous theme in bioinformatics. A typical approach has been the use of a relational database system alongside a web 'front end', allowing deposition and retrieval of content. Where the target data are very structured, comprising lists of homogeneous data, this is an effective strategy with a number of advantages, including rapid retrieval of content. However this approach does not lend itself to collaborative editing of, and addition to the database content in a user-friendly manner, a role for which wikis are uniquely suited.

Biologists are often interested in a specific field and would like to gather and annotate information on biological objects with reference to that field. Collaboration in this limited scope, with deep user derived annotation, can be implemented with wiki technology and is as appropriate as large scale, wide focus wikis. Wikis allow the collborative editing of content by anyone visiting a web page, via simple text markup. Entering the popular conciousness with the advent of Wikipedia http://www.wikipedia.org/, wikis are starting to have an impact in a number of scientific disciplines, fostering communication and collaboration between the members of a research community [1]. Some resources, such as AMYPdb [2], miRDB [3] and RepPop [4] employ wiki technology as part of an interface to a conventional database system. The DOOR database [5] integrates a wiki as a method of facilitating interaction between database users and developers, while the BioPP tool for publishing biological networks [6] provides a wiki as a discussion forum. But perhaps more common is the application of wiki technology to creation of a more generic knowledgebase, similarly to Wikipedia. WikiGenes [7] is a good example of this, and deals specifically with provenance, as a key issue in the application of Wiki technology. LitMiner [8] employs an associated wiki for collaborative editing of the links between genes, tissues, diseases and compounds. The ease of use and collaborative nature of wiki technology has also led to its proposition as a solution to the problem of genome reannotation as knowledge of gene function evolves [9], and to annotation of microarray data [10]. While the collaborative nature of wikis makes them susceptible to error, the principle of 'crowd wisdom', and the in-built version control of many wiki engines offset this limitation [11].

Biological wikis with very broad focus, for example the WikiProfessional concept web [12] can be immensely useful in their unbiased approach to the delineation of diverse, semantically linked concepts. More specialised recent examples are WikiPathways [13] and a gene wiki [14], which cover more specific types of molecular biology data. However the complexity of biological systems is such that the significance of a given gene or protein, for example, can be quite different dependent on research topic. For this reason, the ability to gather together those data pertinent specifically to a system of interest, and annotate them in the context of the system at hand is useful. This can be done in such a way that links to more generic external resources are maintained, allowing the wider research context to be considered where appropriate. Complementary to the generic approach taken by broad-spectrum wikis therefore, there is a role for specialised wikis, with the focus finely tuned to a specific concept. In such a system annotation would be compiled so as to stress the implications for the system of interest, leaving provision of generic information to the linked external resources. Collaborative editing and discussion would be similarly focused.

We present both a generic system to establish base content for a specialised biological wiki, and an example of its use to create a knowledgebase for the renin-angiotensin-aldosterone system, RAASWiki.

## Implementation

The initial goal of this work was to create a comprehensive store of information pertaining to the specific biological system of interest. We decided on a combined approach, starting with seed content easily derived from online resources, to be edited and augmented subsequently by more manual means to maintain a comprehensive, up to date curated resource of current knowledge of the system. BioKb is the set of generically applicable functions developed for this purpose, implemented as a plugin for the TWiki engine.

### Wiki engine

Software to derive initial seed content, which we call BioKB, was developed within the plugin framework of the TWiki engine http://twiki.org/, written in Perl and supplemented with pre-existing JavaScript where appropriate. This approach enables simple integration of BioKb into an existing TWiki installation, and makes the code freely available for critique, and possible development by the community.

Since many other developers have written plugins for TWiki, a number of additional functions are available very simply with minimal extra effort. Features native to TWiki or available in plugins include fine-grained access control, revision control, dynamic embedded search functions and simple incorporation of comments. All of these features could have been constructed independently, but their presence in the TWiki setup significantly eases their incorporation into any wiki-based resource.

TWiki version 4.2 was downloaded from http://twiki.org/. In order to increase speed of search and navigation within the TWiki web where large numbers of topics are present, a pre-existing plugin, variable cache plugin, was applied that allowed caching of topics. This second functionality is especially useful in topics where large amounts of content are generated dynamically: for example subsequent to one or more complex searches. Code to download and compile content, as well as facilitate user input was then written in Perl as a TWiki Plugin, following instructions on the TWiki website. CSS code defining layout and appearance was extensively developed from an example taken from http://www.freecsstemplates.org/preview/grandenally. The use of these modifications to appearance were applied to TWiki by use of the 'pattern' skin of TWiki, enabling simple addition to, or overriding of CSS settings where necessary. All image and CSS files are distributed with the plugin and installed along with the Perl code.

### External information sources

With a KEGG pathway ID as a starting point, the KEGG [15] API is used to obtain details of associated genes, compounds, drugs, and orthologous gene relationships within the pathway. Where appropiate, character strings indicating the organism of origin are incorporated into topic names- for example where the same gene is included from multiple species. Terms suggestive of functional characteristics for genes can be parsed from the MeSH [16] dictionary by use of fuzzy regular expressions, and used as a source of a default textual summary where close matches to MeSH headings were found. Associated synonyms for gene names were obtained from iHOP ([17], http://www.ihop-net.org/). OMIM provided a source for limited details on potential disease associations for genes [18] (with more information available through a link the OMIM page), while relevant publication abstracts are retrieved from PubMed via EUtils http://www.ncbi.nlm.nih.gov/entrez/query/static/eutils_help.html. Employing topic titles and their synonyms, relevant terms in other topics are linked appropriately.

### Provenance

Provenance is an important factor to consider when designing knowledge resources, and has been the subject of significant discussion with users. A simple approach was decided upon, distinguishing between automatic and manually derived annotations. When derived automatically, all fields are tagged with the name of the source database. Where a field is subsequently altered by a user, this is recorded in the metadata for that field, and that user may be consulted for the reason for the change where necessary.

### Available commands

All functions in BioKb are or will be accessible via use of embedded commands in any TWiki system in which the plugin is enabled. For example an input form for molecular biology is placed by default in a topic named 'MolbiolForm', but may also be generated by placing the %MAKE_MOLBIOL_FORM% within a topic. The %MAKE_MOLBIOL% command calls the necessary functions to create a topic directly, when supplied with the correct arguments.

## Results and discussion

### Wiki structure and administration

The objective of BioKb (for 'biological knowledgebase') is to enable creation of specialised knowledgebases benefiting from the complementary features of automatic and collaborative manual annotation. A large number of features were engineered in addition to those available by default in TWiki, including:

• Consistent and automatic formatting of topics of similar type

• Interactive addition of automatically generated topics to a TWiki web based on molecular biology data retrieved by use of a KEGG pathway ID, or paper abstracts produced through a PubMed search

• Post-seeding topic addition via relevant identifiers such as PubMed or gene IDs.

• Assisted topic editing, whereby the more structured content of automatically generated topics is parsed back into an input form for editing, precluding the need to edit wiki code.

• Automatic linking of keywords to topics based on a dictionary relating keywords to topics, also making use of synonyms where appropriate.

The structure of a page in a BioKb wiki consists of two parts. A structured portion at the top allows delineation of fundamental data such as sequence IDs and MeSH terms, and provides links to the source data. A column in this section indicates the origin of the data, and shows if information is derived directly from external resources, or has been created or edited by a user. Functions are provided which allow data in this section to be edited via a form, which both maintains syntax and facilitates the editing process. Underneath the data section, space for unstructured annotation is provided, for example discussion of the importance of a gene for the system(s) of interest. The combination of structured and unstructured input allows for flexible data input, and may reinforce one another. For example, a given group of users might consistently add a particular item to a gene topic- for example the availability of clones etc. Observing this, or at the request of users, an administrator could alter the structure of the data types to include a field for this in future.

**Figure 1:**
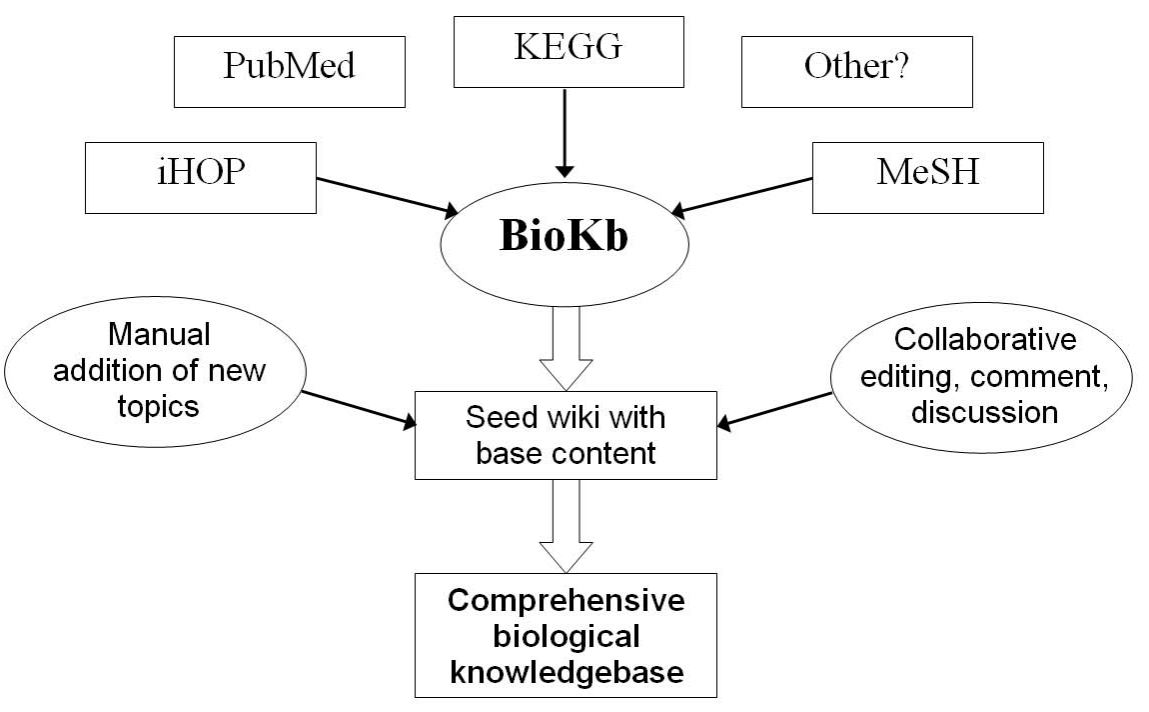
**Conceptual flow for construction of aknowledgebase-wiki with BioKb**. Conceptual flow for construction of a knowledgebase-wiki with BioKb.

Compared to a wiki-only approach, BioKb offers the possibility of added structure- for example summarising the associated IDs and sequences of a gene, as derived from external databases such as KEGG. Relative to a database-driven approach, the inherent features of the underlying wiki engine allow much greater editability and collaboration outside of the structured page regions. Where common themes develop in the unstructured discussions around specific data types, one can imagine these inspiring the addition of new structured elements in the data type. The ability to mix features in this way was a key driver for the development of BioKb, and the usage scheme envisaged is illustrated in Figure 1.

#### Data types

BioKb divides each wiki into three subsections: Disease and Medicine, References, and Molecular Biology, and data types such as gene and disease fall into one of these three categories. Wiki adminstrators can create new data types with a defined set of fields for the structured part of each topic, and assign them to whichever division is most appropriate. For example, data types to represent different high-througput experimental techniques may be incorporated, in addition to 'Gene Expression' present in BioKb by default. This functionality should enable users to extend the usefulness of BioKb webs significantly beyond the default settings.

**Figure 2:**
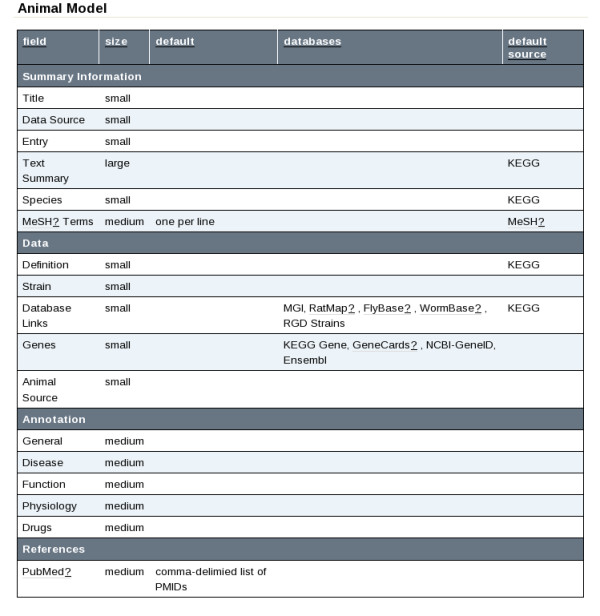
**BioKb administration**. Screenshot showing an excerpt of the BioKb admin topic that defines data content structure in a BioKb-based wiki. From left to right columns define: Field name, a size parameter (defining the type of input field to be used in input forms), default text for input forms, a list of databases which may be referenced by the field, and a default source tag to apply.

BioKb defines a template wiki (or 'web' in the TWiki terminology), from which all BioKb wikis are derived. Some of the pages (or 'topics') defined in this wiki determine the appearance of a given data type (for example 'Reference'). Others provide summary sections for each data type, and are created with embedded TWiki search functionality that will provide overviews of content once the wiki is populated. 'Administration' topics, also provided by default, can be used to alter the functionality that BioKb provides over that present in TWiki, and can be customised prior to the population of a wiki. Most important of these is a definition of the data type and form structure, illustrated in Figure 2. This topic is referenced whenever an input form is generated or a new molecular biology topic is created with BioKb functions, and can be adjusted by wiki administrators. Fields are defined and assigned to particular types of topic (gene, compound etc), and category (summary, data, annotation) within topics. Field order is significant, such that topics will be created with fields displayed in the order they are defined within the admin topic. This topic also allows administrators to define fields that require auxillary drop-down fields, and an example present by default is a list of databases pertinent to fields that request a database ID. The drop-down is displayed beside the ID field, with the option to add more pairs of fields- for example when referring to many databases. The root URLs into which IDs are insered in order to reference external databases are also provided via an admin topic, along with a function that allows for updating of these URLs in such a way that pre-existing examples are revised accordingly. Other admin topics include a definition of which organisms to include when examining orthologous genes.

### Wiki population

BioKb provides four complementary ways to populate a wiki, with varying requirements for user input. On creation of a BioKb web, users are presented with default content including summary pages and lists of topics of various types. Embedded functions, for example those placed by default in the left toolbar, can then be used to incorporate content by manual or automatic means as described below.

#### Large-scale automatic based on pathway definitions

By supplying a KEGG pathway ID, a list of associated genes (and their orthologs), compounds, drugs, disease states (derived from a search of OMIM's [18] 'morbid map' of disease-associated genes) and references are presented. Some or all of these may be selected for input to the wiki to form intial seed content. This KEGG-based seeding was the simplest method available, but options employing references to other databases will be incorporated in future development. KEGG-derived genes are supplemented with synonyms from iHOP before being incorporated into topics. Functions are provided in some topics for retrieval of information too time-consuming to incorporate during the seeding process- for example relevant MeSH terms. Each field is clearly tagged with the date, and resource and/or individual from which it is derived.

#### Free text

Wiki technology empowers the users of web-based resources by allowing them to make changes to content rapidly through use of a simplified markup language, an advantage that drove our adoption of wiki methods for this work. We provide a number of template topics with BioKb that can be selected at topic creation in order apply CSS parameters appropriate to the topic type (e.g. molecular biology, disease and medicine) while allowing free content creation. This might not be appropriate where a structured data type is required, for example listing the properties of a gene, but might be in other situations where a conceptual data type and associated fields are difficult to define.

#### Form based

Topics may be created by the use of forms generated dynamically following definitions in an Admin topic. By completing the fields in this form, the result is a topic structure identical to that achieved by automated means, thus maintaining consistency. The fields in this form can also be populated on-screen if an external ID (e.g. KEGG Gene ID) is supplied, allowing the user to edit the information from these resources prior to topic creation. The form also contains fields for input of identifiers pertaining to external databases. In contrast to purely manual topic creation, these IDs are presented to BioKb in a structured way, so that appropriate URLs can be constructed. In order to track provenance of the data, where fields are not altered from the source, source and date alone are recorded. Where a user has edited the field, this is indicated. A function is provided in each such structured data section to read out the information into a form again for editing. This method has the advantage that non-technical users do not encounter wiki syntax.

#### Interactive selection of topics for automatic addition

Another available function is a form to enable complex queries to PubMed. Similar to the seeding process, a user is presented with a list of articles and titles, alongside an indication of whether they currently form part of the wiki. Users may choose to add or remove paper abstracts from the list presented to them for incorporation. Since references are not mutable over time, users select which to include, but are not presented with the opportunity to edit them via form functions- though this can be achieved through the native wiki functions.

### Cross-linking of topics

On topic creation by any of the means described above, a reference dictionary of key terms and topics is consulted, and used to add cross-links where appropriate. This dictionary is also amended with details of the topic currently under construction, to allow links to it on future topic creation events, and links added to it from all pre-existing topics based on keyword matching and use of appropriate iHOP-derived synonyms. To reduce the occurrence of incorrect links, linked terms must be longer than three characters, and the dictionary is accessible as an admin function, to allow corrections where topics are linked inappropriately to particular terms. Where a term matches to multiple dictionary terms, the longest match is selected to preserve specificity.

### Automatic maintenance of content

BioKb is distributed with scripts which, when added to the task scheduling system of an operating system, can be used to update content regularly. For example abstracts can be regularly retrieved from PubMed corresponding to a specific search string, and its keywords linked appropriately to the rest of the wiki. We do not, however, envisage regular automatic updates of the seed content of existing topics. In a knowledgebase of this kind it is important that consistency is mainted between manual and automatic annotations. The types of annotation derived at seeding are fairly static (links to external databases etc), but where revisions to seed data are necessary, we would expect this to be undertaken manually, with the help of the form editing functions provided, preserving manual annotations.

### The results of BioKb are intended as a framework for further annotation

BioKb is not intended simply as an aggregator of existing online resources, but rather as a method by which a framework for collaboration within a research group may be established. Remote queries establish basic information such as protein sequence, synonyms and possible disease associations, around which further annotation and discussion may be placed in a more manual, but possibly more informative way. As in all wiki approaches, we would hope that user groups will used seed data, and expand upon it, with only administrative or structural pages o3 limits for editing.

### RAASWiki: An exemplar Wiki knowledgebase built on a foundation created with BioKB

The renin-angiotensin-aldosterone system (RAAS) is a molecular pathway implicated in a number of physiological systems, most notably in the regulation of blood pressure and fluid balance. A number of diseases are associated with disruption of RAAS function, and manipulation of the RAAS is often the first target of therapeutic drugs against high blood pressure (hypertension). This system is highly relevant to many research groups worldwide, including those in our institute, and the collation (and collaborative curation) of information relevant to this system was therefore highly desirable.

A focused scope for a specific field allows a biologist to concentrate on immediately relevant information for that gene. Of course, the relevance will be defined by the user or group. A user of RAASWiki is likely to be interested in the involvement of membrane metallo-endopeptidase in hypertension, but less so in its role in Alzheimer's. Similarly, cathepsin G has many interactions outside the RAAS that might be less relevant to a user of RAASWiki.

Using the BioKb system, we engineered a knowledgebase for the RAAS (RAASWiki, http://www.raaswiki.org). The KEGG database contains an entry for the renin-angiotensin system (ID hsa04614; http://www.genome.ad.jp/kegg/pathway/hsa/hsa04614.html), and this served as the starting point in construction of RAASWiki. However the KEGG pathway does not incorporate other genes tightly associated with the pathway, most importantly those associated with aldosterone, and these were incorporated piece-wise by use of BioKb's form interface. In addition, the illustration used on the front page of RAASWiki was custom-designed by use of 'Wordle' [19]. Due to lack of availability through automated means, a number of topics with details on animal models were created manually through the form interface (described above). These additions illustrate some of the ways in which a wiki based on BioKb can be improved upon by users through incremental additions.

Similarly to other biological wikis and databases, it is clearly important that a biological object (gene for example) is unambiguously identified in RAASWiki. We have attempted to use a fairly well accepted standard, the official gene symbol to identify a gene. In addition, other database identifiers (Entrez gene, Ensembl) are given where available. Hyperlinks using these identifiers allow the user to navigate to many alternative resources for each gene. Literature references are identified by PubMed ID; Gene expression dataset by GEO or Array Express identifier and so on. These automatically generated links replicate how a biologist would navigate the available information.

**Figure 3:**
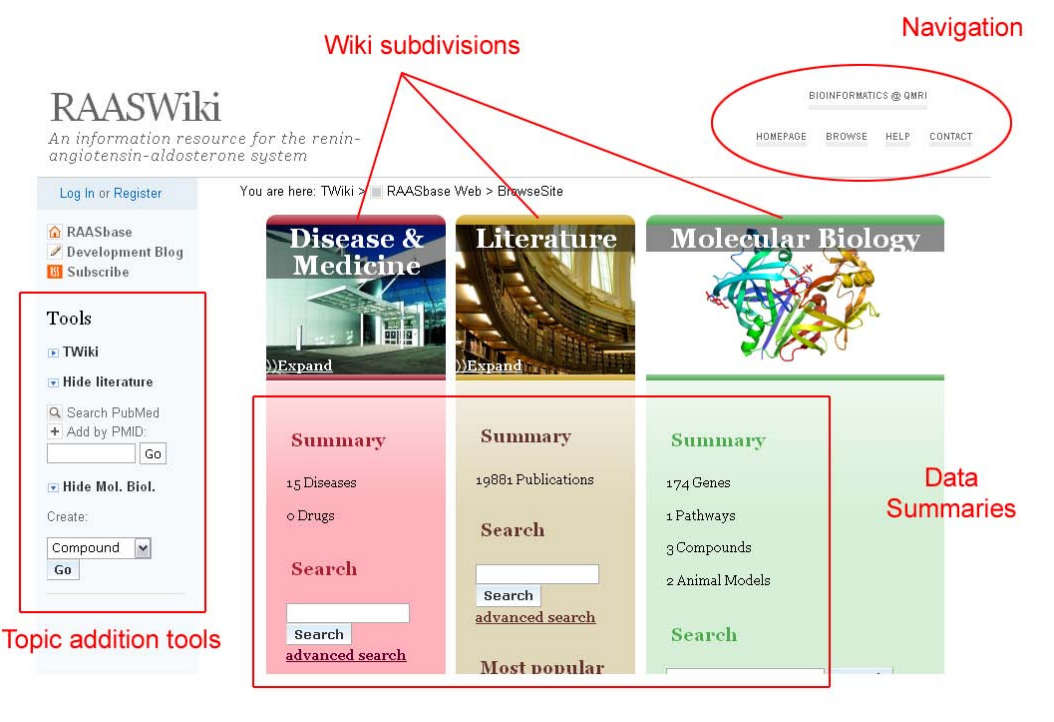
**Screenshot showing overview screen from RAASWiki**. Screenshot showing overview screen from RAASWiki.

Figure 3 shows the 'Browse' screen of RAASWiki. This page contains a large number of embedded searches that provide statistics on the current status of the wiki, making the page-caching functionality of a TWiki plugin extremely useful. At the time of submission, RAASWiki contained 21 human genes (with 29 associated orthologous groups); 3 pathways; 80 compounds; 11 animal models; 5 diseases; 27 drugs; and 10,460 abstracts containing hyperlinks to molecular biology data topics wherever possible.

### A further wiki example

Another pathway of significance to our institute is that of leukocyte transendothelial migration. As an example of the versatility of the BioKB system, we also created LTEMWiki http://www.bioinf.mvm.ed.ac.uk/twiki/bin/view/LTEMWiki. This was generated by reference to the pathway definition for leukocyte transendothelial migration (pathway ID hsa04670) found in KEGG [15], and has not yet been further adapted beyond generation of a front-page image.

### User feedback and future development

The core philosophy of RAASWiki, and more generally of BioKb, is the use of automatically derived data as core around which to assemble information not easily obtained by automatic means, in particular data and annotation specific to a system of interest. User participation will be of central importance and development is therefore ongoing for both BioKb and RAASWiki with input from laboratory scientists.

#### BioKb

• **A more diverse range of sources for automatically derived seed data **Predominant among planned developments for BioKb is the incorporation of as many sources as possible for seed content and automatic population of input forms.

• **Provenance **Users stressed the importance of knowing the origins of data displayed. A simple provenance scheme has been implemented (see above), but other aspects will be developed- for example to indicate the reasoning associated with including a given publication in the wiki (in a similar, but more focused way to the 'Faculty of 1000').

• **More integral provision for discussion **The ability to undertake discussions around the data present in a wiki is highly useful, and we will be examining ways of improving upon this. The current implementation allows discussion via a series of comments in a given topic in a discussion-board style. It may be that discussion common to a set of topics would be useful, for example in a threaded 'forum' style, and the possibilities for this will be investigated.

#### RAASWiki

User feedback on RAASWiki from laboratory researchers has indicated a number of areas for development, many of which are ongoing, and may feed back into BioKb development. RAASWiki will be developed in the next phase of work, in collaboration with laboratory scientists, illustrating the intended use of automatic content as a foundation for high-quality manual additions.

• **Documentation and examples **It was suggested that a more explicit delineation of the goals of RAASWiki would be useful to users and contributors. We have taken a first step here by placing some introductory content on RAASWiki's front page, and in a new 'about' page. However, it will be necessary to 'flesh out' a small subset of topics in each type, on selected genes and publications for example, in order to suggest the type of content envisaged. This will involve some collaborative effort with laboratory scientists, but with more exposure it is our hope that community annotation will build on the foundation present, moving RAASWiki beyond a set of aggregated of data. This process, from a foundation of automatically derived data to a collaboratively annotated resource, is also the scenario in which we would expect other users to employ BioKb.

• **Further data types **One comment made was that the highly focused nature of this type of wiki makes it an ideal place to store laboratory-related data difficult to find elsewhere. An example is animal models, which are already present, but could perhaps be more extensive, and play a more central role in the resource. Other topic types are under consideration- for example for system-specific protocols and reagents. Addition of such data, together with links to the relevant publications, and associated comments on usefulness, will form an important component of RAASWiki development. The expanding set of data types may have implications for the organisation of topics, as represented on the 'Browse' page of RAASWiki, and possibilities for reorganisation will be explored.

## Conclusion

For the creation of a set of base data for a comprehensive knowledgebase on the renin-angiotensin-aldosterone system, we developed a software utility that may be used to automatically seed a wiki with easily available information on molecular biology, references, and disease associated with a given biological pathway. We present here both this software utility and one outcome from it: the RAASWiki knowledgebase, and it is our hope that other biological researchers can make similar use of BioKb. Through the assistive functions of BioKb we aim to expand the content of RAASWiki to provide a comprehensive resource on this clinically very significant system.

The type of wiki we have discussed is very focused: BioKb is designed to allow semi-manual annotation centred around a very specific biological system, tuned to the needs of individual groups of researchers. This approach is complementary to large-scale resources covering, for example, the entirety of known proteins, pathways, or interactions, and we ancipate the two variants would be most useful in concert.

## Availability and requirements

• Project name: BioKb

• Project Home page: http://www.bioinf.mvm.ed.ac.uk/twiki/bin/view/TWiki/BioKbPlugin

• Operating systems: Platform independent (subject to TWiki installation)

• Programming language: Perl

• Other requirements: TWiki version 4.2, TWiki plugins VotePlugin, PatternSkin, TwistyPlugin and AccessStatsPlugin (plus associated dependencies), perl modules XML::Simple, XML::LibXML, LWP::Simple, SOAP::Lite 0.60 and Clone (all available on CPAN)

• License: GPL

## Authors' contributions

DRD and JJM conceived the original ideas for this work; implementation and development was carried out by JRM, AH carried out work to supply some of the content for RAASWiki. The manuscript was prepared by JRM and DRD with input from AH. All authors read and approved the final manuscript.
